# Disparities in Current Cigarette Smoking Among US Adults With Mental Health Conditions

**DOI:** 10.5888/pcd19.220184

**Published:** 2022-12-22

**Authors:** Caitlin G. Loretan, Teresa W. Wang, Christina V. Watson, Ahmed Jamal

**Affiliations:** 1 Office on Smoking and Health, National Center for Chronic Disease Prevention and Health Promotion, Centers for Disease Control and Prevention, Atlanta, Georgia

## Abstract

**Introduction:**

Prevalence of cigarette smoking is disproportionally high among US adults with mental health conditions. Adults with mental health conditions who smoke cigarettes are at increased risk for smoking-related illness and death compared with adults without mental health conditions.

**Methods:**

We analyzed pooled data from the 2019 and 2020 National Survey on Drug Use and Health to provide national estimates of current cigarette smoking prevalence among US adults aged 18 years or older who reported having in the past year any mental illness, serious mental illness, mild or moderate mental illness, serious psychological distress, and/or major depressive episode (N = 19,398) and state-level estimates for any mental illness.

**Results:**

Prevalence of cigarette smoking for serious mental illness was 27.2%; serious psychological distress and major depressive disorder, 25.0%; serious psychological distress, 24.5%; any mental illness, 22.8%; mild or moderate mental illness, 21.2%; and major depressive disorder, 17.6%. State-level cigarette smoking prevalence among adults with any mental illness ranged from 11.7% in Utah to 42.1% in Louisiana, with a median of 24.7%.

**Conclusion:**

The prevalence of current cigarette smoking is higher among adults with any mental illness, psychological distress, and major depressive disorder than among those without any mental illness, especially among adults who are non-Hispanic American Indian or Alaska Native, Hispanic, lesbian, gay, or bisexual and among those who are experiencing poverty, are uninsured, or have been arrested and booked in the past year. Continued improvement in integration of smoking cessation interventions into mental health treatment, equitable implementation of comprehensive commercial tobacco control policies, and population-specific approaches could reduce cigarette smoking among adults with mental health conditions.

**SUMMARY**

**What is already known on this topic?**

The prevalence of cigarette smoking is higher among US adults with mental health conditions than adults without mental health conditions.

**What is added by this report?**

During 2019–2020, more than 1 in 5 adults with any past-year mental illness said they smoked. Disparities in the prevalence of cigarette smoking among adults with mental illness exist at the state level.

**What are the implications for public health practice?**

Our study helps identify populations who could benefit from improved integration of smoking cessation interventions into mental health treatment and equitable implementation of comprehensive commercial tobacco control policies to prevent smoking initiation and increase smoking cessation among adults with mental health conditions.

**MEDSCAPE CME**

In support of improving patient care, this activity has been planned and implemented by Medscape, LLC and *Preventing Chronic Disease*. Medscape, LLC is jointly accredited with commendation by the Accreditation Council for Continuing Medical Education (ACCME), the Accreditation Council for Pharmacy Education (ACPE), and the American Nurses Credentialing Center (ANCC), to provide continuing education for the healthcare team.

Medscape, LLC designates this Journal-based CME activity for a maximum of 1.0 AMA PRA Category 1 Credit(s)™. Physicians should claim only the credit commensurate with the extent of their participation in the activity.

Successful completion of this CME activity, which includes participation in the evaluation component, enables the participant to earn up to 1.0 MOC points in the American Board of Internal Medicine’s (ABIM) Maintenance of Certification (MOC) program. Participants will earn MOC points equivalent to the amount of CME credits claimed for the activity. It is the CME activity provider’s responsibility to submit participant completion information to ACCME for the purpose of granting ABIM MOC credit.

**Release date: December 22, 2022; Expiration date: December 22, 2023**

**Learning Objectives**

Upon completion of this activity, participants will:

1. Compare the prevalence of cigarette smoking among US adults with mental illness vs those without mental illness
2. Distinguish types of mental illness particularly associated with cigarette smoking
3. Identify the racial/ethnic group with the highest prevalence of cigarette smoking
4. Assess variables associated with higher rates of cigarette smoking among adults with mental illness

**Credit Hours –1.0**

**EDITOR**

Rosemarie Perrin

Editor

*Preventing Chronic Disease*

Atlanta, GA

**AUTHORS**

Caitlin G. Loretan, MPH

Centers for Disease Control and Prevention

Atlanta, Georgia

Teresa W. Wang, PhD

Centers for Disease Control and Prevention

Atlanta, Georgia

Christina V. Watson, DrPH

Centers for Disease Control and Prevention

Atlanta, Georgia

Ahmed Jamal, MBBS

Centers for Disease Control and Prevention

Atlanta, Georgia

**CME AUTHOR**

Charles P. Vega, MD

Health Sciences Clinical Professor of Family Medicine

University of California, Irvine School of Medicine

Charles P. Vega, MD, has the following relevant financial relationships:

Consultant or advisor for: GlaxoSmithKline; Johnson & Johnson Pharmaceutical Research & Development, L.L.C.

## Introduction

Approximately 1 in 5 adults in the US lives with or experiences a mental health condition each year. These same people are more likely to smoke cigarettes than the population overall (1,2). Adults with mental health conditions who smoke cigarettes are at increased risk for smoking-related illness and death compared with adults without mental health conditions (3). Of the approximately 480,000 annual deaths attributable to tobacco products, about 200,000 are among adults who smoke and have a mental health condition (4). Higher rates of cigarette smoking among adults with mental health conditions is a result of many factors, including targeting by the tobacco industry (5).

Previously reported estimates of cigarette smoking prevalence among adults with mental health conditions were 40.8% for serious psychological distress (2016) (6), 33.3% for any mental illness (AMI) in the past year (2012–2014) (7), 30.5% for major depressive disorder (2022) (8), and 26.9% for regular feelings of depression (2020) (9). The national smoking prevalence among all US adults, regardless of mental health condition, was 12.5% in 2020 (9). Prior research has found disparities in cigarette smoking among adults with AMI. Higher smoking prevalence was identified among males, people aged 18 to 49 years, non-Hispanic American Indian and Alaska Native people, people with less than a high school education, people living in poverty, unemployed people, and residents of the Midwest (2,7).

Recently, declines in cigarette smoking among adults with mental health conditions have been reported (8,10); however, prevalence remains high relative to those without mental health conditions (6–8). Ongoing surveillance and reporting of recent cigarette smoking estimates among adults with mental health conditions is needed to continue to monitor trends and help identify sociodemographic groups that could benefit from improved tobacco screening, smoking cessation interventions, and mental health treatment (10). Limited information exists in the literature regarding state-level estimates of cigarette smoking among adults with mental health conditions, although evidence indicates that prevalence varies by region (2). The most recent comprehensive estimates were reported from 2009 through 2011 (2). By using data from the 2019–2020 National Survey on Drug Use and Health (NSDUH), we examined the prevalence of past 30-day cigarette smoking (current smoking) among adults with past-year mental health conditions and the prevalence by state of current smoking among adults aged 18 years or older with and without AMI.

## Methods

### Study sample

NSDUH is an annual, national household survey of the civilian, noninstitutionalized US population aged 12 years or older. Data are collected through a combination of interviewer-administered questions and computer-assisted self-administered questions (11). Our analysis was restricted to adults aged 18 years or older. Data were pooled across the 2019 and 2020 survey years to account for small sample sizes in stratified analysis. Participants who did not respond to the mental health questions or provide smoking status (n = 4), mental health condition questions alone (n = 301), or smoking status alone (n = 18) were excluded from analysis. We conducted our analysis from 2021 through 2022.

### Mental health conditions

We based definitions of all mental health conditions on recoded variables in the NSDUH data set. Only those variables found in NSDUH were included in our analysis; therefore, we could not calculate current smoking prevalence for categories in the anxiety domain alone. Detailed NSDUH definitions of mental health disorders are available (11). Because our focus was on mental health, substance use was not explored separately from mental health (8).

### Mental illness

Past-year AMI was defined as having a diagnosable mental, behavioral, or emotional disorder other than developmental or substance use disorder, based on the *Diagnostic and Statistical Manual of Mental Disorders–IV* (11). Serious mental illness, defined as a subset of estimates of AMI, was limited to participants with mental, behavioral, or emotional disorders that interfered substantially or limited one or more major life activities (eg, basic daily living skills; instrumental living skills; functioning in social, familial, and vocational context) with a Global Assessment of Functioning (GAF) score of ≤50 (11). Mild or moderate mental illness was defined as having AMI in the past year and reporting mild to moderate impairment and a GAF score of ≥51 (12). Serious mental illness and mild or moderate mental illness were included in AMI; however, serious mental illness and mild or moderate mental illness were mutually exclusive categories.

### Psychological distress

Past-year serious psychological distress was defined as a having a score of 13 or more of 24 on the Kessler Psychological Distress scale (eg, feeling nervous, feeling hopeless, feeling restless, feeling sad or depressed, feeling that everything was an effort, feeling down on yourself) during the month in the past year when they were at their worst emotionally (11).

### Major depressive disorder

Past-year major depressive episode — major depressive disorder — was based on having been classified as having had a major depressive episode in one’s lifetime and having experienced a period of depression or loss of interest or pleasure in daily activities lasting 2 weeks or longer in the past 12 months (11).

A person with serious psychological distress or major depressive disorder could be classified in any mental illness category (ie, serious mental illness or mild or moderate mental illness plus AMI). No mental illness reported in the past year included anyone who did not report AMI, serious psychological distress, or major depressive disorder in the past year.

### Measures

Current smoking was defined as having smoked 100 or more cigarettes in one’s lifetime and smoking at least part or all of a cigarette in the 30 days preceding the interview. We calculated prevalence estimates overall for the past year for current smoking among adults with no AMI, serious psychological distress, or major depressive disorder in the past year; AMI; serious mental illness; mild or moderate mental illness; serious psychological distress alone; major depressive disorder alone; and both serious psychological distress and major depressive disorder. Estimates were further stratified by measures that included survey year, age, sex, sexual orientation, race or ethnicity, education, disability, annual household income, marital status, health insurance, employment status, metropolitan statistical area, and having been arrested and booked in the past year. State-level prevalence of current smoking by AMI status was reported by using 2-year (2019–2020) data from NSDUH’s restricted-use data analysis system (https://rdas.samhsa.gov/#/).

### Statistical analysis

Data were weighted to adjust for survey nonresponse and to provide national and state-level representative estimates. We calculated prevalence estimates and corresponding symmetrical 95% CIs. Extent of disparity (absolute disparity) was calculated by using the absolute difference in the prevalence of smoking by AMI. Estimates with relative standard error greater than 30% were suppressed. SAS-callable SUDAAN software 11.0.3 (RTI International) was used to conduct all analyses.

## Results

The NSDUH adult survey response rate was 64.2% in 2019 and 62.8% in 2020 (11). An unweighted sample of 68,427 adults aged 18 years or older completed interviews in the combined 2019 and 2020 survey years. Our primary study population consisted of 19,398 (28.3%) surveyed adults aged 18 years or older who reported AMI, serious psychological distress, or major depressive disorder in the past year. Of those, 4,251 (21.9%) currently smoked. A total of 6,896 (10.1%) reported no AMI, serious psychological distress, or major depressive disorder and currently smoked. The populations for the state-level analysis were taken directly from NSDUH’s restricted-use data analysis system; therefore, unweighted sample sizes are unknown.

The national prevalence of current smoking during 2019–2020 was 27.2% among adults with serious mental illness, 25.0% among adults with serious psychological distress and major depressive disorder, 24.5% among adults with serious psychological distress alone, 22.8% among adults with AMI, 21.2% among adults with mild or moderate mental illness, and 17.6% among adults with major depressive disorder alone (Table 1). The prevalence of current cigarette smoking among almost all mental health conditions was relatively higher than among adults without a mental health condition, except among a few demographic subgroups reporting serious psychological distress, major depressive disorder, or both. The absolute disparity in current smoking by mental health condition was highest among non-Hispanic American Indian and Alaska Native (non-Hispanic AI/AN) adults with serious psychological distress (45.0%), serious mental illness (33.2%), mild or moderate mental illness (24.8%), or AMI (27.3%); adults with less than a high school diploma and serious mental illness (25.2%) or serious psychological distress and major depressive disorder (20.5%); uninsured adults with serious mental illness (20.7%); and adults arrested and booked in the past 12 months with major depressive disorder (26.1%), serious mental illness (25.8%), or serious psychological distress (22.1%).

**Table 1:** Percentage of Adults Who Currently Smoke Cigarettes, by Mental Health Condition, National Survey on Drug Use and Health, 2019–2020

Characteristic	Current cigarette smoking^a^						
	No past-year mental health condition (n = 6,896)	Past-year any mental illness^b ^(n = 3,983)	Past-year serious mental illness (n = 1,336)	Past-year mild or moderate mental illness (n = 2,647)	Past-year serious psychological distress alone (n = 1,498)	Past-year major depressive disorder alone (n = 303)	Past-year serious psychological distress and major depressive disorder (n = 1,337)
**Overall**	13.6 (13.0–14.3)	22.8 (21.7–23.8)	27.2 (25.3–29.0)	21.2 (19.8–22.6)	24.5 (22.3–26.7)	17.6 (13.8–21.5)	25.0 (23.3–26.8)
**Survey year**							
2019	14.6 (14.0–15.2)	25.2 (24.3–26.2)	30.1 (27.7–32.6)	23.6 (22.4–24.8)	27.7 (25.7–29.6)	18.5 (15.6–21.4)	27.2 (25.3–29.2)
2020	12.7 (11.7–13.7)	20.3 (18.4–22.1)	24.3 (20.9–27.7)	18.9 (16.3–21.4)	21.3 (17.3–25.3)	16.7 (9.4–24.0)	22.9 (20.0–25.9)
**Age, y**							
18–25	9.7 (8.9–10.5)	14.9 (13.8–16.0)	18.9 (16.6–21.2)	13.1 (11.8–14.5)	14.3 (12.4–16.3)	10.6 (7.6–13.6)	16.2 (14.1–18.3)
26–34	16.5 (15.6–17.4)	26.1 (24.4–27.9)	31.6 (27.9–35.4)	24.1 (22.3–26.0)	26.7 (23.3–30.1)	25.1 (15.7–34.6)	30.2 (26.2–34.1)
35–49	17.4 (16.4–18.3)	27.8 (26.0–29.6)	34.4 (30.5–38.3)	25.3 (23.3–27.4)	31.6 (28.0–35.2)	21.5 (15.9–27.2)	32.1 (27.9–36.3)
50–64	15.7 (14.4–17.1)	27.0 (23.4–30.6)	27.0 (21.6–32.3)	27.0 (22.3–31.7)	32.1 (22.3–41.9)	15.9 (10.1–21.7)	28.7 (22.2–35.1)
≥65	7.7 (6.7–8.8)	11.6 (8.4–14.7)	— c	11.8 (8.3–15.4)	18.1 (8.8–27.4)	— c	11.8 (4.9–18.6)
**Sex**							
Male	15.8 (15.0–16.6)	25.5 (23.8–27.1)	29.1 (26.0–32.1)	24.3 (22.2–26.3)	30.1 (26.2–34.0)	16.4 (12.2–20.6)	28.0 (25.0–31.0)
Female	11.4 (10.6–12.2)	21.2 (19.8–22.5)	26.1 (23.6–28.6)	19.4 (17.6–21.2)	20.7 (18.7–22.7)	18.4 (13.0–23.7)	23.3 (20.8–25.9)
**Sexual orientation**							
Heterosexual	13.4 (12.7–14.0)	22.1 (21.0–23.3)	27.1 (24.9–29.3)	20.6 (19.1–22.2)	24.1 (21.8–26.3)	18.1 (13.9–22.2)	24.7 (22.8–26.6)
Gay/lesbian	20.7 (16.1–25.2)	29.2 (23.8–34.6)	29.3 (19.1–39.5)	29.2 (21.6–36.7)	29.4 (20.6–38.2)	— c	31.3 (21.0–41.7)
Bisexual	22.3 (19.5–25.0)	25.9 (22.9–28.9)	28.5 (24.9–32.2)	23.9 (20.0–27.8)	26.8 (22.4–31.2)	17.1 (9.6–24.5)	25.4 (21.6–29.1)
**Race/ethnicity**							
Hispanic	8.5 (7.6–9.4)	19.0 (16.0–22.1)	23.2 (17.8–28.5)	17.7 (14.1–21.3)	21.7 (14.7–28.8)	12.8 (6.1–19.5)	18.6 (14.2–22.9)
Non-Hispanic American Indian, Alaska Native	26.0 (18.8–33.2)	53.3 (40.5–66.2)	59.2 (34.5–83.8)	50.8 (33.8–67.8)	71.0 (50.1–92.0)	— c	40.8 (19.2–62.4)
Non-Hispanic Asian	7.2 (5.1–9.2)	7.9 (4.5–11.3)	— c	8.1 (4.2–11.9)	6.0 (2.8–9.2)	— c	— c
Non-Hispanic Black	16.2 (14.2–18.2)	21.0 (16.9–25.0)	17.4 (11.0–23.9)	22.1 (17.6–26.6)	24.8 (18.7–30.9)	— c	15.9 (10.5–21.4)
Non-Hispanic multiple races	23.4 (18.1–28.6)	29.1 (22.6–35.5)	32.5 (22.8–42.3)	27.9 (19.3–36.4)	22.8 (11.9–33.7)	23.0 (10.1–35.9)	26.4 (18.5–34.4)
Non-Hispanic Native Hawaiian, Pacific Islander	13.5 (8.3–18.7)	— c	— c	— c	— c	— c	— c
Non-Hispanic White	14.8 (13.9–15.7)	24.1 (22.8–25.3)	29.6 (27.4–31.9)	22.1 (20.4–23.8)	26.0 (23.4–28.6)	18.6 (13.8–23.5)	28.0 (26.2–29.9)
**Education**							
Less than high school diploma	21.1 (19.2–23.1)	36.3 (31.2–41.4)	46.3 (37.0–55.5)	33.8 (28.2–39.3)	33.2 (24.8–41.7)	— c	41.6 (33.6–49.7)
High school diploma/GED	19.0 (17.5–20.4)	32.3 (29.5–35.2)	33.0 (28.4–37.7)	32.0 (28.7–35.4)	37.7 (33.2–42.3)	29.8 (20.4–39.3)	31.5 (26.9–36.1)
Some college/associates degree	14.8 (13.8–15.8)	23.1 (21.6–24.6)	26.3 (23.5–29.1)	21.7 (19.6–23.9)	22.2 (19.1–25.4)	16.8 (12.5–21.0)	24.2 (21.5–27.0)
College graduate	5.6 (5.1–6.2)	10.5 (9.3–11.7)	16.9 (14.2–19.5)	8.7 (7.6–9.9)	9.6 (7.5–11.8)	10.1 (6.5–13.8)	14.7 (12.1–17.4)
**Disability^d^**							
Yes	15.4 (13.5–17.3)	26.7 (24.9–28.5)	29.8 (26.8–32.7)	24.9 (22.5–27.4)	27.8 (24.5–31.0)	23.1 (15.6–30.6)	29.6 (26.5–32.8)
No	13.3 (12.7–13.9)	19.8 (18.5–21.2)	23.9 (21.0–26.7)	18.9 (17.2–20.6)	22.7 (20.1–25.4)	13.9 (10.5–17.3)	19.8 (17.6–21.9)
**Annual household income**							
Income at or below federal poverty threshold	22.5 (20.4–24.7)	36.7 (33.6–39.7)	37.6 (32.0–43.2)	36.2 (32.5–40.0)	36.8 (31.3–42.3)	24.8 (14.5–35.1)	35.9 (31.0–40.9)
Income up to 2x federal poverty threshold	18.5 (16.9–20.1)	28.1 (25.9–30.4)	32.4 (28.1–36.7)	26.6 (23.7–29.4)	26.9 (22.9–30.8)	27.8 (15.8–39.9)	29.6 (26.2–33.1)
Income more than 2x federal poverty threshold	10.9 (10.3–11.5)	17.0 (15.9–18.1)	21.4 (19.0–23.8)	15.6 (14.3–16.9)	19.4 (16.5–22.3)	12.9 (9.9–15.9)	19.5 (17.2–21.9)
**Marriage status**							
Married/living with partner	10.0 (9.2–10.8)	17.7 (15.9–19.5)	22.1 (18.6–25.6)	16.4 (14.5–18.4)	21.2 (17.1–25.4)	15.7 (9.9–21.5)	20.3 (16.9–23.8)
Divorced/separated/widowed	19.6 (17.7–21.6)	30.2 (27.9–32.6)	36.7 (32.1–41.3)	27.8 (24.9–30.7)	35.8 (29.3–42.3)	21.1 (14.8–27.4)	37.9 (32.6–43.3)
Never married	17.1 (16.1–18.1)	23.4 (22.2–24.6)	25.9 (23.3–28.4)	22.4 (20.9–23.9)	22.4 (20.4–24.4)	17.5 (12.4–22.6)	22.5 (20.4–24.7)
**Health insurance^e^**							
Public	18.5 (16.9–20.1)	31.6 (29.2–33.9)	32.7 (28.8–36.6)	31.1 (27.9–34.2)	32.7 (28.9–36.4)	26.5 (15.8–37.1)	31.7 (27.9–35.5)
Private	10.5 (9.9–11.1)	15.7 (14.6–16.8)	19.8 (17.1–22.4)	14.5 (13.2–15.8)	17.3 (14.4–20.2)	13.1 (10.3–15.9)	17.3 (15.0–19.6)
Uninsured	23.1 (21.2–25.0)	37.7 (34.4–40.9)	43.8 (37.3–50.3)	35.2 (31.5–38.9)	37.7 (32.9–42.5)	24.8 (12.7–36.9)	40.0 (34.6–45.4)
**Employment status**							
Full time	14.4 (13.7–15.2)	20.5 (19.1–22.0)	25.6 (23.3–27.9)	19.0 (17.3–20.6)	22.0 (19.4–24.7)	16.0 (11.2–20.9)	23.7 (21.1–26.2)
Part time	11.6 (10.3–12.9)	16.4 (14.4–18.4)	20.0 (15.7–24.3)	15.1 (12.9–17.2)	14.6 (10.9–18.3)	13.8 (8.4–19.3)	17.9 (14.0–21.8)
Unemployed	23.3 (20.3–26.2)	35.7 (31.3–40.1)	36.8 (27.6–46.1)	35.2 (29.6–40.8)	35.4 (29.3–41.4)	29.2 (13.7–44.6)	32.2 (25.9–38.4)
Other/not in labor force	12.1 (11.1–13.2)	26.3 (23.6–28.9)	30.3 (26.6–34.1)	24.8 (21.4–28.1)	30.2 (25.2–35.3)	19.1 (11.1–27.1)	28.8 (24.9–32.7)
**Metropolitan statistical area^f^**							
Large metro	11.6 (10.8–12.4)	19.3 (18.0–20.7)	22.9 (20.1–25.8)	18.2 (16.4–20.0)	20.7 (17.3–24.0)	16.7 (10.4–23.1)	20.4 (17.8–23.1)
Small metro	15.5 (14.5–16.5)	24.6 (22.7–26.4)	30.1 (26.9–33.3)	22.4 (19.8–25.0)	26.6 (22.8–30.3)	16.3 (12.3–20.3)	28.3 (25.3–31.3)
Nonmetro/rural	17.6 (16.2–18.9)	31.3 (28.3–34.3)	34.0 (28.4–39.5)	30.3 (26.7–34.0)	33.9 (29.2–38.7)	24.4 (15.6–33.3)	33.2 (27.8–38.7)
**Arrested and booked in past 12 months**							
Yes	44.1 (38.9–49.2)	63.0 (56.8–69.2)	69.9 (60.8–78.9)	59.3 (51.4–67.2)	66.2 (53.2–79.2)	70.2 (43.4–96.9)	62.1 (52.1–72.1)
No	30.3 (28.8–31.8)	40.5 (38.2–42.7)	41.5 (36.1–46.8)	40.0 (37.2–42.8)	47.4 (42.0–52.7)	21.2 (15.7–26.6)	44.7 (38.5–51.0)

Abbreviations: GED, General Educational Development.

a Current cigarette smoking was defined as respondents who smoked ≥100 cigarettes in their lifetime and reported smoking part or all of a cigarette in the 30 days preceding interview. Values are weighted percentage (95% CI).

b Past year any mental illness was defined as respondents who reported serious, moderate, or mild mental illness, serious psychological distress, or a major depressive disorder in the past year.

c Estimates suppressed because relative standard error was >30%.

d Disability was defined as respondents reporting any of the following: deaf or difficulty hearing; blind or serious difficulty seeing, even when wearing glasses; serious difficulty concentrating, remembering, or making decisions because of a physical, mental, or emotional condition; serious difficulty walking or climbing stairs; difficulty dressing or bathing; difficulty doing errands alone such as visiting a doctor’s office or shopping because of a physical, mental, or emotional condition.

e Public and private health insurances are not mutually exclusive; public insurance includes Medicaid, Child Health Improvement Plan, Medicare, Tricare, Champus, Veterans Administration, or some other military insurance.

f Metropolitan statistical areas are based on the 2013 Rural–Urban Continuum Codes (www.ers.usda.gov/data-products/rural-urban-continuum-codes.aspx).

State-specific current smoking prevalence among adults with AMI was consistently higher than among adults without AMI (Table 2). By state, cigarette smoking prevalence among adults with AMI ranged from 11.7% in Utah to 42.1% in Louisiana, with a median of 24.7% in South Carolina. State-level absolute disparity in current smoking by AMI ranged from 1.6% in the District of Columbia to 21.9% in Louisiana (Table 2).

**Table 2:** Percentage of Adults Who Currently Smoke Cigarettes, by State and Mental Illness Status, National Survey on Drug Use and Health, 2019–2020^a^

State	AMI^b^	AMI and smoke cigarettes^b^^,^ c	No AMI and smoke cigarettes^c^^,^ d
State median, %	21.4	24.7	16.0
Alabama	20.5 (16.7–25.0)	35.0 (25.8–45.6)	19.9 (15.4–25.4)
Alaska	21.1 (17.5–25.3)	22.5 (15.5–31.6)	16.4 (13.0–20.4)
Arizona	24.1 (20.7–27.8)	16.4 (11.2–23.4)	12.6 (9.2–17.1)
Arkansas	22.3 (18.3–26.9)	35.3 (26.8–44.8)	18.3 (14.1–23.3)
California	20.1 (18.6–21.7)	19.1 (16.0–22.6)	11.2 (9.6–13.0)
Colorado	23.3 (19.9–27.0)	20.7 (14.7–28.3)	12.2 (9.3–15.9)
Connecticut	15.9 (13.1–19.2)	18.6 (12.7–26.3)	11.3 (8.0–15.8)
Delaware	20.8 (17.3–24.7)	24.2 (18.4–31.1)	17.0 (12.6–22.4)
District of Columbia	23.7 (20.2–27.7)	19.8 (13.6–27.8)	18.2 (13.5–24.2)
Florida	16.9 (15.2–18.7)	23.1 (18.8–28.0)	15.9 (13.9–18.2)
Georgia	15.7 (13.2–18.5)	20.0 (14.1–27.6)	15.3 (12.4–18.8)
Hawaii	17.9 (14.6–21.8)	22.6 (15.5–31.8)	15.6 (12.0–20.1)
Idaho	24.9 (21.5–28.6)	21.1 (15.3–28.3)	11.8 (8.2–16.7)
Illinois	20.1 (18.0–22.4)	23.9 (19.1–29.3)	15.3 (13.2–17.8)
Indiana	20.6 (17.2–24.5)	35.0 (28.6–42.0)	20.1 (16.1–24.9)
Iowa	19.7 (16.0–24.0)	26.4 (19.4–34.9)	17.7 (13.5–22.8)
Kansas	28.5 (24.3–33.1)	25.4 (17.9–34.6)	16.3 (12.3–21.5)
Kentucky	21.6 (18.1–25.6)	34.2 (26.5–42.9)	22.3 (18.2–26.9)
Louisiana	21.1 (17.8–24.9)	42.1 (32.2–52.7)	20.2 (16.4–24.5)
Maine	22.0 (17.6–27.1)	23.8 (15.7–34.4)	15.0 (10.9–20.4)
Maryland	16.6 (14.1–19.5)	23.4 (17.6–30.3)	11.5 (8.8–14.8)
Massachusetts	21.4 (17.2–26.3)	18.6 (10.8–30.2)	11.7 (9.6–14.2)
Michigan	22.7 (20.3–25.4)	27.9 (23.7–32.6)	18.1 (15.5–21.2)
Minnesota	23.6 (20.8–26.7)	28.4 (21.3–36.8)	10.5 (8.4–13.2)
Mississippi	22.1 (18.4–26.3)	32.4 (22.8–43.7)	22.6 (18.5–27.4)
Missouri	19.8 (17.0–22.9)	29.6 (23.2–36.9)	16.4 (13.2–20.1)
Montana	23.0 (18.8–27.9)	28.6 (21.3–37.1)	19.1 (16.4–22.1)
Nebraska	23.0 (20.1–26.2)	30.5 (21.4–41.3)	12.3 (9.5–15.9)
Nevada	21.2 (17.2–25.9)	26.7 (19.7–35.1)	11.9 (8.9–15.9)
New Hampshire	26.3 (22.1–31.0)	22.0 (15.7–29.9)	13.8 (10.7–17.6)
New Jersey	17.8 (15.1–20.8)	17.9 (12.8–24.5)	12.1 (9.7–15.1)
New Mexico	21.6 (17.6–26.1)	25.2 (18.6–33.1)	15.5 (10.8–21.8)
New York	18.7 (17.0–20.6)	25.6 (21.6–30.0)	14.0 (12.2–15.9)
North Carolina	18.6 (16.8–20.6)	27.0 (20.7–34.3)	17.9 (15.3–20.8)
North Dakota	18.8 (16.2–21.8)	29.0 (21.2–38.2)	18.6 (14.6–23.4)
Ohio	24.8 (22.1–27.7)	34.2 (29.2–39.5)	19.1 (16.5–22.1)
Oklahoma	29.1 (25.2–33.3)	28.7 (22.0–36.5)	21.4 (17.0–26.5)
Oregon	27.5 (23.7–31.7)	21.5 (15.3–29.3)	13.8 (10.8–17.4)
Pennsylvania	19.5 (16.9–22.3)	29.5 (23.1–36.8)	18.9 (15.5–22.8)
Rhode Island	23.9 (20.2–28.1)	20.8 (13.3–31.0)	16.2 (12.1–21.4)
South Carolina	23.3 (19.5–27.5)	24.7 (18.7–31.9)	19.9 (16.8–23.5)
South Dakota	18.4 (15.7–21.5)	28.6 (19.5–39.8)	19.4 (14.5–25.5)
Tennessee	19.6 (16.2–23.6)	32.2 (25.3–40.0)	18.0 (14.1–22.7)
Texas	17.5 (15.7–19.4)	23.0 (18.9–27.7)	14.7 (12.9–16.6)
Utah	31.5 (27.8–35.5)	11.7 (7.9–17.1)	7.8 (6.1–10.0)
Vermont	22.8 (19.1–26.9)	27.4 (20.2–36.0)	10.4 (8.0–13.5)
Virginia	20.4 (17.8–23.4)	18.9 (13.7–25.5)	15.2 (12.4–18.4)
Washington	26.1 (22.4–30.1)	20.1 (15.1–26.3)	15.4 (12.3–19.0)
West Virginia	28.3 (24.1–32.9)	31.6 (23.2–41.4)	20.7 (16.2–26.2)
Wisconsin	21.1 (18.2–24.2)	22.1 (16.9–28.3)	16.0 (12.0–20.9)
Wyoming	24.1 (20.3–28.3)	25.1 (17.7–34.1)	19.7 (14.7–25.9)

Abbreviation: AMI, any mental illness.

a Values are weighted percentage (95% CI) unless otherwise indicated.

b Any mental illness was defined as a participant who reported serious, moderate, or mild mental illness, serious psychological distress, and/or major depressive disorder in the past year.

c Current cigarette smoking was defined as those reporting smoking part or all of a cigarette in the past 30 days before interview.

d No serious, moderate, or mild mental illness, serious psychological distress, or major depressive episode reported over the past year.

## Discussion

During 2019–2020, the prevalence of current cigarette smoking remained high among adults with mental health conditions. Overall, the proportion of current smoking among adults with AMI was approximately 67% higher than among those without AMI. Disparities in current smoking among subpopulations with mental health conditions were similar to disparities in current smoking among adults without mental health conditions, albeit with generally higher current smoking prevalence (9). Previously reported higher prevalence of current smoking among subpopulations of adults with mental health conditions are still apparent, including among adults aged 26 to 64 years; those who are lesbian, gay, or bisexual (LGB), or non-Hispanic AI/AN; have less than a high school diploma; have an annual income below the federal poverty threshold; are uninsured; live in a rural area; or have been arrested and booked in the past 12 months. Between-state variations also exist. These findings suggest it is possible that adults with mental health conditions may continue to experience greater smoking initiation or barriers to successful smoking cessation.

Several possible factors related to smoking cessation could be associated with high smoking prevalence among adults with mental health conditions. Barriers to providing smoking cessation services exist in mental health care settings and among mental health clinicians, including misconceptions about the effect of smoking on behavioral health conditions and their treatment (4). More than half of psychiatric providers believe their patients are not interested in quitting; in contrast, among a sample of adults who smoked and were hospitalized with mental illness (4), almost 2 of 3 participants were interested in quitting, suggesting that adults who smoke and have mental illness are motivated to quit (12). Smoking cessation is associated with reduced depression, anxiety, and posttraumatic stress disorder (PTSD) symptoms, whereas continued smoking is associated with elevated levels of anxiety and depressive symptoms (4,13). An additional barrier to reducing cigarette smoking among adults with mental health conditions who are treated in mental health facilities is a lack of tobacco-free grounds. As of January 1, 2022, 14 states required tobacco-free grounds for most mental health facilities (14). These results are based on legal requirements made in each state. Additional behavioral health facilities can and do take voluntary action on creating tobacco-free grounds (14). Adults with mental health conditions may have more severe dependence on commercial tobacco and nicotine than those who do not have a mental health condition, so intensive interventions that use a range of evidence-based cessation services, including pharmacotherapy and counseling, are important to improve cessation success (5). Recent studies have found declines in cigarette smoking among adults with mental health conditions, although not to the same extent as among adults without mental health conditions (8,10).

Current smoking was high among adults of all racial and ethnic groups with AMI relative to no AMI. However, the greatest difference among adults with and without AMI was seen among Hispanic and non-Hispanic AI/AN adults. Cigarette smoking among Hispanic adults tends to be lower than among other racial and ethnic groups (9). We found a larger disparity in current smoking among Hispanic adults with and without AMI than previously reported (2). It is unclear why smoking is more prevalent among Hispanic adults with AMI, although this finding is partially supported by findings that indicated use of mental health services — as a general proxy for psychiatric comorbidity — was significantly associated with smoking among a small group of Hispanic adults (15).

Historically, non-Hispanic AI/AN adults have had among the highest rates of cigarette smoking (not including traditional ceremonial tobacco use) of all racial and ethnic groups in the US (16). We found current smoking estimates were twice as high among non-Hispanic AI/AN adults with AMI than among those without (53% vs 26%), with almost 1 of 2 non-Hispanic AI/AN adults with a mental health condition reporting current smoking (16). Although the sample size for non-Hispanic AI/AN adults was small, we found that those with serious psychological distress had one of the highest rates of cigarette smoking of all demographic groups. non-Hispanic AI/AN adults may have experiences of current and historic trauma, including PTSD, high rates of violent victimization, and overall higher rates of mental health conditions compared with other racial and ethnic groups (17). These factors, in addition to past policies that have led to mistrust of government services and care, such as removal from their land, high rates of poverty, barriers to appropriate health care and mental health services, and an already high prevalence of current smoking may be related to the high prevalence of current smoking among non-Hispanic AI/AN adults who have mental health conditions (17,18). Trauma-informed care and culturally appropriate smoking cessation resources for non-Hispanic AI/AN adults may be important considerations when developing or providing cessation interventions for this population.

Smoking prevalence is high in the US among adults who identify as LGB. In 2020, current smoking prevalence was 16.1% among LGB adults, compared with 12.3% among those who identified as heterosexual (9). Our results are consistent with these findings. The tobacco industry has historically advertised heavily and promoted commercial tobacco products to the LGB community, especially in bars and clubs (19). High prevalence of commercial tobacco use among LGB adults suggests that these strategies were successful (19). Adults in the LGB and other sexual orientation and gender identity minority communities may experience stress caused by concealment of sexual orientation and expectation of rejection and may be more likely to be subjected to bullying, all of which have the potential to cause or exacerbate mental illness (20,21). Through these internal and external stressors, a person may turn to coping mechanisms, which could include smoking (20). To help reduce cigarette smoking among LGB adults with mental health conditions, access to mental health care providers with appropriate knowledge of LGB health and with the use of evidence-based cessation interventions could help reduce this disparity (21).

Adults who were arrested and booked in the last 12 months and reported AMI, serious mental illness, mild or moderate mental illness, serious psychological distress alone, major depressive disorder alone, or both were among those with the highest prevalence of current smoking in our study. Almost 3 out of 4 people who had been arrested and had serious mental illness or major depressive disorder reported current smoking. The act of being arrested can adversely affect a person, regardless of whether they are convicted or sentenced (22). An arrest could result in a label of “criminal,” which can limit a person’s opportunities and increase or exacerbate stress (22). In turn, this may lead to stress-coping behaviors such as smoking (22). People in prisons and jails are disproportionately affected by mental illness, with more than 50% experiencing mental illness in any given year (23). Collaboration between criminal justice, public health, and mental health researchers could provide opportunities for further research into managing mental illness and reducing cigarette smoking among those who are incarcerated, adoption of smoke-free jails and prisons, and resources such as providing mental health care and access to smoking cessation services to people immediately upon incarceration (23).

Our study generally found lower levels of cigarette smoking among adults with the assessed mental health conditions compared with previously reported estimates (6–9). However, our study was not designed to examine significant changes in cigarette smoking among these populations over time. The use of different sources of data (9) and use of different methodologies to calculate estimates may also contribute to variations in reported estimates (8). Future examination of estimates among this population over time using the same sources of data are warranted to accurately detect trends and point out increases or decreases in cigarette smoking prevalence.

State-level estimates of current smoking were consistent with, but generally lower than, previous findings (2). Commercial tobacco and smoke-free laws and policies vary by state and locality, and approximately 40% of the US population are not covered by comprehensive smoke-free laws for workplaces, restaurants, and bars (24). Smoke-free policies have been shown to reduce cigarette consumption, increase cessation attempts, and increase rates of successful cessation (25). Additionally, states have variable mental health care systems — for example, 55% of US counties do not have a practicing psychiatrist — as well as inconsistent commercial tobacco-related regulations, including varying reimbursement rates for providing smoking cessation services (26,27). The results of our study suggest that, although smoking prevalence by state among adults with AMI is lower than previously reported, there is still room for improvement.

Very limited information exists on smoking prevalence by state among people with AMI or other mental health conditions. The results of a study conducted in New York State indicated that tobacco retailer density was independently and positively associated with smoking among adults with comorbid diabetes and serious mental illness (28). The authors’ reported that smoking cessation support was readily available in the state mental health system, yet many patients continued to smoke (28). Some populations may benefit from further commercial tobacco–related protections and public health interventions, such as equitable implementation of comprehensive commercial tobacco control policies, improved integration of smoking cessation interventions into mental health treatment facilities, and other population-specific approaches.

### Limitations

The findings of our study are subject to limitations. First, mental health conditions in the past year and current smoking are self-reported and may be affected by self-report and social desirability bias. Race and ethnicity may be related to underestimates of mental health conditions. Lower prevalence of depression among non-Hispanic Black and Hispanic adults than among non-Hispanic White adults may reflect self-report differences rather than true differences (29). However, estimates for AMI, serious mental illness, and mild or moderate mental illness were based on a previously developed statistical model that was itself based on responses to the Kessler Psychological Distress questions (11) and depression-related questions — not just a yes/no indicator question — which may have helped limit self-report bias. Second, NSDUH does not include adults in its sampling frame who were, at the time of survey, institutionalized, incarcerated, or on active military duty; therefore, results cannot be generalized to these populations. However, a weighted count of 1,570,874 adults in the 2019–2020 NSDUH reported on AMI in the past year and reported an overnight stay in a hospital for mental health treatment by a positive response to the question, “During the past 12 months, have you stayed overnight or longer in a hospital or other facility to receive treatment or counseling for any problem you were having with your emotions, nerves, or mental health? Please do not include treatment for alcohol or drug use.” Therefore, NSDUH may capture results from some people with a prior history of brief institutionalization. Lastly, we were unable to make statistical comparisons in state-level current smoking prevalence among adults with and without AMI because of restrictions on unweighted state data. Strengths of this study include providing updated national estimates of current smoking among noninstitutionalized adults with mental health conditions in the past year and state-level estimates of current smoking among adults with AMI.

### Conclusion

Current cigarette smoking remains higher among adults with AMI, serious psychological distress, and major depressive disorder than among those without AMI, especially among adults who are Hispanic, non-Hispanic AI/AN, or LGB and populations experiencing poverty, lack of health insurance, or were arrested and booked in the past year. Our study adds further evidence that adults with mental health conditions may be disproportionately affected by multiple external factors that lead to ongoing high prevalence of current smoking. The findings from this article contribute to our knowledge of cigarette smoking disparities, especially among adults with mental health conditions and cigarette smoking by US state. Addressing cigarette smoking among adults with mental health conditions may require interventions that can reduce barriers particular to this population, in addition to the equitable implementation of well-established comprehensive commercial tobacco prevention and control strategies. Population-specific approaches could include education on evidence-based practices for treating people who smoke and have a mental health condition, increasing the number of smoke-free mental health treatment facilities, and improving access to cessation services, particularly within the criminal justice system.

## Post-Test Information

To obtain credit, you should first read the journal article. After reading the article, you should be able to answer the following, related, multiple-choice questions. To complete the questions (with a minimum 75% passing score) and earn continuing medical education (CME) credit, please go to http://www.medscape.org/journal/pcd. Credit cannot be obtained for tests completed on paper, although you may use the worksheet below to keep a record of your answers.

You must be a registered user on http://www.medscape.org. If you are not registered on http://www.medscape.org, please click on the “Register” link on the right hand side of the website.

Only one answer is correct for each question. Once you successfully answer all post-test questions, you will be able to view and/or print your certificate. For questions regarding this activity, contact the accredited provider, CME@medscape.net. For technical assistance, contact CME@medscape.net. American Medical Association’s Physician’s Recognition Award (AMA PRA) credits are accepted in the US as evidence of participation in CME activities. For further information on this award, please go to https://www.ama-assn.org. The AMA has determined that physicians not licensed in the US who participate in this CME activity are eligible for AMA PRA Category 1 Credits™. Through agreements that the AMA has made with agencies in some countries, AMA PRA credit may be acceptable as evidence of participation in CME activities. If you are not licensed in the US, please complete the questions online, print the AMA PRA CME credit certificate, and present it to your national medical association for review.

## Post-Test Questions

### Study Title: Disparities in Current Cigarette Smoking Among US Adults With Mental Health Conditions

#### CME Questions

1. The prevalence of cigarette smoking was what percentage higher in comparing the populations with vs without any mental illness in the current study?
  - 12%
  - 67%
  - 110%
  - 315%
2. Which diagnostic cohort of adults with mental illness had the highest prevalence of cigarette smoking in the current study?
  - Major depression alone
  - Major depression plus serious psychological distress
  - Any mental illness
  - Serious psychological distress alone
3. Which racial/ethnic group had the highest rates of cigarette smoking in the current study?
  - Native Americans/Alaska Natives
  - Black
  - Hispanic
  - Hawaiian/Pacific Islander
4. Which of the following variables in the current study was most associated with higher disparities in cigarette smoking prevalence among adults with mental illness?
  - Arrest and booking and lack of health insurance
  - Being male and urban environment
  - Some college educational attainment and being single
  - Being female and identifying as LGBT
